# Refinement of Different Frequency Bands of Geomagnetic Vertical Intensity Polarization Anomalies before M > 5.5 Earthquakes

**DOI:** 10.3390/s24103240

**Published:** 2024-05-20

**Authors:** Haris Faheem, Xia Li, Weiling Zhu, Yingfeng Ji, Lili Feng, Ye Zhu

**Affiliations:** 1 State Key Laboratory of the Tibetan Plateau Earth System, Environment and Resources (TPESER), Institute of Tibetan Plateau Research, Chinese Academy of Sciences, Beijing 100101, China; haris@itpcas.ac.cn (H.F.); lxqhdz@163.com (X.L.); zhuweiling@itpcas.ac.cn (W.Z.); zhuye@itpcas.ac.cn (Y.Z.); 2 University of Chinese Academy of Sciences, Beijing 100049, China; 3 Qinghai Earthquake Administration, Xining 810001, China; dzj350102@163.com

**Keywords:** frequency bands, geomagnetic vertical intensity polarization, earthquake prediction

## Abstract

Geomagnetic vertical intensity polarization is a method with a clear mechanism, mature processing methods, and a strong ability to extract anomalous information in the quantitative analysis of seismogenic geomagnetic disturbances. The existing analyses of geomagnetic vertical intensity polarization are all based on the 5~100 s frequency band without refinement of the partitioning process. Although many successful results have been obtained, there are still two problems in the process of extracting anomalies: the geomagnetic anomalies that satisfy the determination criteria are still high in occurrence frequency; and the anomalies are distributed over too large an area in space, which leads to difficulties in determining the location of the epicenter. In this study, based on observations from western China, where fluxgate observation points are positioned in areas with frequent, densely distributed medium-strength earthquakes, we refined the frequency bands of geomagnetic vertical intensity polarization, recalculated the spatial and temporal evolution characteristics of geomagnetic disturbances before earthquakes, and improved the crossover frequency anomaly prediction index while promoting the application of the method in earthquake forecasting.

## 1. Introduction

In recent years, electromagnetic waves with ultralow frequency (ULF) signal anomalies have been identified during rock fractures (e.g., [1,2]). Experimental studies suggest that ULF (0.01–10 Hz) electromagnetic radiation is indeed generated during the rock rupture process in zero magnetic space (e.g., [3,4,5]). Short-term precursory changes in the magnetic field in rocks occur near the time of rupture [3]. The discovery of anomalous ULF band electromagnetic (EM) signals prior to the 1988 Spitak earthquake and the 1989 Loma Prieta earthquake has attracted extensive attention from scientists worldwide (e.g., [6,7,8,9]). Molchanov and Hayakawa [10] discussed the physical mechanism of preseismic EM radiation anomalies through rock rupture experiments. From the point of view that currents are generated during microrupture and after simple assumptions about dilatation and stick–slip instability, those researchers confirmed that rocks generate ULF EM anomalous signals during rock rupture. They further utilized many numerical simulations to demonstrate that the amplitude of the vertical component of the magnetic signal from the intracrustal frequency near 1 Hz is greater than the amplitude of the horizontal component of the primary source, and its ratio is greater than 1. Therefore, the ratio of the two can be utilized to suppress the electromagnetic signal from the exogenous field while highlighting the anomalous signal of the lithosphere (e.g., [10]). Hayakawa, et al. [11] analyzed the polarization characteristics before and after the 8.0 magnitude Guam earthquake on 8 August 1993, using observation data from a fluxgate magnetometer at the Geomagnetic Station in Guam, United States, which was located 65 km from the epicenter of the earthquake. They found that the polarization in the 0.01–0.05 Hz frequency band increased gradually in the two months prior to the earthquake, reached a maximum at the time of the earthquake, and recovered gradually after the earthquake. In Japan, a five-year project (April 1997–March 2002) was carried out in the central part of the East China Sea to establish a station array with several fluxgate observation sites, during which ULF anomalous signals were observed before four earthquakes and a cluster of events [12]. In 1999, Russian and Japanese scientists established an integrated geophysical observatory in the seismically active area of the Karimshino Peninsula, Russia. They carried out a comprehensive study of seismic anomalies, including the extraction of ULF electromagnetic anomalous signals, which were also detected prior to several earthquakes (e.g., [13]).

However, Thomas, et al. [14] compared their data with 1 s data from the Kakioka observatory in Japan and the global magnetic activity index Kp and found an absence of identifiable localized anomalous signals occurring prior to the earthquake proposed by Hayakawa, Kawate, Molchanov and Yumoto [11]. Changes in polarization are part of normal global magnetic activity and are unrelated to earthquakes [14]. By re-examining the magnetic precursor data reported by Fraser-Smith, Bernardi, McGill, Ladd, Helliwell and Villard Jr [6] at the time of the Loma Prieta earthquake, Thomas, Love and Johnston [14] inferred that the key components of the precursory signal identified can be explained by minor corruption of the data in the form of a gain enhancement and time-stamp misassignment, possibly due to digital processing errors or inadvertent postacquisitional treatment. The fractal behaviors of previously reported seismogenic ULF magnetic signatures depend mainly on geomagnetic activity due to solar-terrestrial interactions [15].

Among the methods for extracting short-period geomagnetic anomaly signals, vertical intensity polarization has a clear mechanism, a mature processing method, and a strong ability to extract anomalous information (e.g., [16]) and has been used in pre-earthquake geomagnetic anomaly extraction because it is a promising method for earthquake prediction (e.g., [1,11]). This method is based on spectral analysis, which compares the spectral amplitudes of the vertical component Z and the horizontal component (H or G) of the lithospheric magnetic field, thus enabling people to distinguish lithospheric ULF electromagnetic signals from ULF electromagnetic signals originating from the ionosphere (e.g., [3]). In recent years, the geomagnetic vertical intensity polarization method has become a hotspot of research in China, and a series of earthquakes, such as the 2004~2007 Kashgar earthquake [17], the 2009 Binchuan M5.0 earthquake [18], the 2017 Jiuzhaigou M7.0 and Jinghe M6.6 earthquakes [19], and the 2017 Alashan M5.0 earthquake [20], were found to be associated with significant anomalous seismomagnetic signals. However, there are still two problems in the process of extracting anomalies: first, the frequency of anomalies that satisfy the determination criteria is too high; second, the anomalies are spatially distributed over too large an area, which leads to difficulties in determining the location of the epicenter. The key reason for the above problems is that the selected frequency bands have a wide range (5~100 s), and further refinement of the partitioning of the EM frequency has not been analyzed in depth.

According to the skin effect of electromagnetism theory, a lower-frequency electromagnetic field has a greater penetration depth, and the electromagnetic radiation signal varies with different seismic mechanisms, tectonic environments, fault geometries, and crustal attenuation, resulting in frequency ranges with different patterns. Moreover, due to the different sensitivities of each observational instrument to anomalies, interference anomalies are easily confused with seismic precursor anomalies. Therefore, by refining the frequency band of geomagnetic vertical intensity polarization, we can not only obtain anomalous changes in shallow media but also filter out some of the short-period interference effects and improve the utilization rate of data to enhance the effectiveness of this method in earthquake prediction.

Numerous case studies have shown that high polarization anomalies in the 0.01 Hz (100 s) band were observed from several days to three months before earthquakes [21,22,23,24,25,26,27,28,29]. However, from the skin effect, it is known that the frequency range of seismomagnetic disturbances varies for earthquakes with different source depths and different seismic tectonics, and not all preseismic anomalies are concentrated in the 0.01 Hz (100 s) frequency band. Therefore, Hobara, et al. [30] performed a finer frequency segmentation of the polarization results and extracted more significant anomalous signals of the preseismic polarization values of the 1997 Guam 8.0 earthquake and the 2000 Izu earthquake swarm in the frequency bands of 0.02–0.022 Hz (45–50 s) and 0.05–0.1 Hz (10–20 s), respectively. Moreover, based on fluctuation theory, a simple three-dimensional anomaly body model is established, and the seismic magnetic perturbation signals coinciding with the anomalous polarization values are obtained via numerical simulation (e.g., [30]). By using second-sampling observation data from five stations of the China Geomagnetic Observatory Network, Li, et al. [31] found that the ULF anomaly with a frequency band of 0.1 Hz (10 s) is more appropriate for detecting earthquake precursors and estimating hypocentral depth according to skin effects. The anomaly amplitude was significantly enhanced half a month before the earthquake, and the amplitude was inversely proportional to the epicentral distance [31]. Hence, the geomagnetic vertical intensity polarization method has good practical application, is in line with electromagnetic signal propagation theory, and has in-depth research significance. The extraction of geomagnetic field information by this method has received widespread attention and has become a research hotspot.

## 2. Methods

In this study, we mainly target and focus on the 0.01–0.2 Hz (5–100 s) frequency band of geomagnetic measurements and its refinement. The digitized observation data from geomagnetic stations across the country, which started on 1 January 2002, are updated on a daily basis. The components of the geomagnetic data include geomagnetic H, geomagnetic Z, declination D, declination I, fluxgate X, fluxgate Y, fluxgate Z, and fluxgate temperature. The format of the data is the international IAGA-2002 format, which includes the station name, IAGA code, longitude and distance, and temperature. The data are in the common international IAGA-2002 format, including station name, IAGA code, latitude, longitude, elevation, measurement component, adoption rate, data type, time-averaged value of each element of D, H, Z, F, and annual average value of each of the seven elements of D, H, Z, F, X, Y, and I. The data are also in the IAGA-2002 format. To obtain geomagnetic data, CTM-302 three-component high-resolution fluxgate magnetometers are widely employed in the China Geomagnetic Observatory Network. The magnetometer accusation system includes an MP430 control unit, a real-time clock, a three-component geomagnetic signal A/D analog-to-digital conversion unit, a three-channel D/A digital-to-analog conversion unit, a temperature/inclination measurement unit, and an RS-422 communication unit. The MSP430 control unit is based on the RISC structure of a 16-bit mixed-signal processor (e.g., [32]). We mainly emphasize the vertical intensity polarization values, which are defined by the ratio of the spectral value of the geomagnetic vertical component to that of the horizontal component at a measurement point. Moreover, the calculation steps involved processing the data, which included the division of daily second sampling data into sections, calculation of spectra for specific frequency bands, and residual analysis and smoothing techniques applied to enhance the accuracy of the data processing.

Based on the full-frequency band (5~100 s) analysis of geomagnetic vertical intensity polarization, we redivided the data from the computation period of 5~100 s into three frequency bands (5~25 s, 25~50 s, and 50~100 s) and extracted the seismogenic magnetic disturbance anomalies. At the same time, we collected geomagnetic data preceding M > 5 earthquakes in the study area, analyzed the anomaly characteristics and their relationships with earthquakes, and extracted the dominant frequency bands.

The geomagnetic vertical intensity polarization value (*Y_zh_*) is defined as the ratio of the spectral value of the geomagnetic vertical component to that of the horizontal component at a measurement point.

(1) $$Y_{zh}=\left|\frac{Z(\omega )}{H(\omega )}\right|$$

(2) $$H(\omega )=\sqrt{H_{x}^{2}(\omega )+H_{y}^{2}(\omega )}$$

Here, *Z*(*ω*) is the spectral value of the geomagnetic vertical component, *H*(*ω*) is the spectral value of the geomagnetic horizontal component, *H_x_*(*ω*) is the north–south spectral value of the geomagnetic horizontal component, and *H_y_*(*ω*) is the east–west spectral value of the geomagnetic horizontal component. The unit of each component is nanotesla (nT). The calculation steps include the following:

(1) Assuming 15 min to be a section, the daily second sampling data are divided into 96 sections, and the spectra of the periods 5~25 s, 25~50 s and 50~100 s and their vertical intensity polarization amplitude values, as well as the daily average values, are calculated;
(2) The fuzzy fitting curves are calculated with the daily average values of the vertical intensity polarization with a period of ≥183 days as well as the variance of their residuals;
(3) The fuzzy fitting curve plus two times the variance of its residuals is taken as the threshold line; then, the frequency points where the amplitude of the vertical intensity polarization is lower than the threshold (the external air field influence) are excluded, and the daily mean of the polarization values is recalculated;
(4) Fuzzy fitting is performed with the daily average values of the vertical intensity polarization with a period of ≥183 days, and the residuals of the daily mean are calculated. The 5-day sliding average of the residuals is calculated to eliminate the high-frequency effect.

## 3. Results

**Figure 1:**
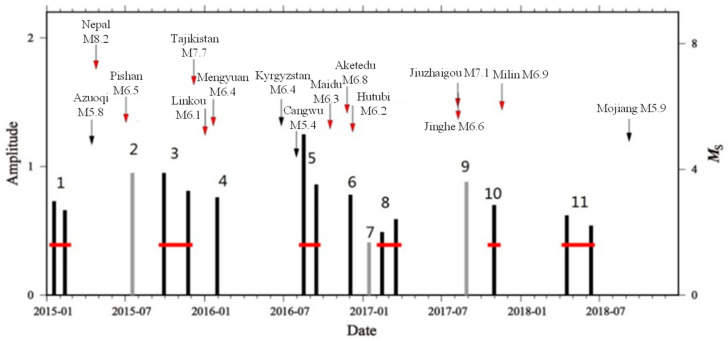
Characteristics of geomagnetic vertical intensity polarization anomalies in groups (full frequency band: 5~100 s). The black vertical lines in the figure are precursory anomalies, and the gray vertical lines are postearthquake effects. Red bars indicate the amplitude threshold.

Based on the full-frequency band analysis and the earthquake cases, a total of eleven groups of anomalies, including sixteen single-day anomalies and five superimposed anomalies, were calculated, and the frequency band processing was refined and divided into 5~25 s, 25~50 s, and 50~100 s. Then, the anomalous features were further sorted, and the spatial variations in the geomagnetic vertical intensity polarization anomalies in the different frequency bands are shown in Figure 1 and Table 1.

By combining the calculation results from 2015, the spatial distribution of anomalies in January and February shows that although the four earthquakes corresponding to the full-frequency band in one year (the Menyuan M6.4 earthquake, the Azuoqi M5.8 earthquake, the Pishan M6.5 earthquake, and the Nepal M8.2 earthquake) were located in the area with anomalously high values, including almost the entire western part of China, by refining the frequency band into the 25~50 s and 50~50 s domains, the significant anomalies are reduced in area; moreover, the correspondence of anomalies to certain seismic events becomes clearer.

**Figure 2:**
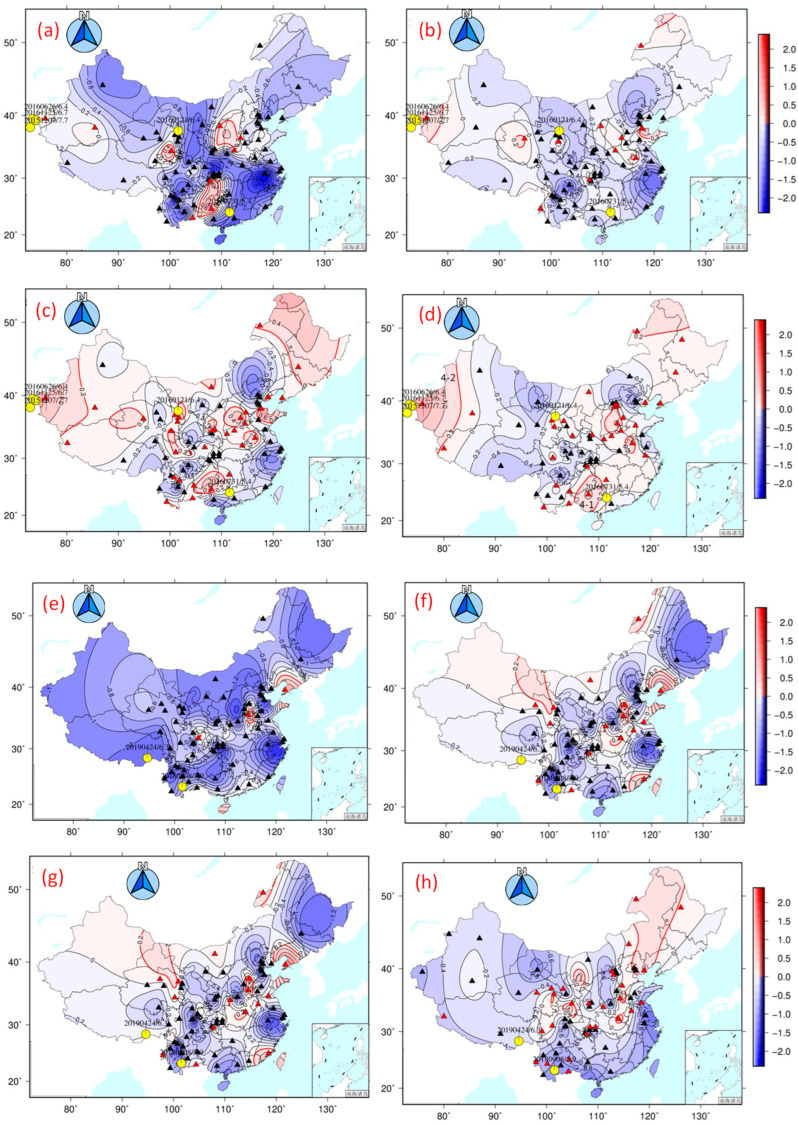
Spatial variation in geomagnetic vertical intensity polarization anomalies in different frequency bands before M > 5 earthquakes. Red triangles indicate the stations with observed anomalies. The black triangles indicate all of the stations used in this study. Yellow circles indicate the epicenters of M > 5.5 earthquakes after the observed anomalies. (**a**–**d**) On 30 January 2016, before the 2016 Cangwu Guangxi M5.4 earthquake. (**a**) 5~25 s; (**b**) 25~50 s; (**c**) 50~100 s; (**d**) 5~100 s. (**e**,**f**) On 12 June 2018, before the 2018 Mojiang 5.9 earthquake. (**e**) 5~25 s; (**f**) 25~50 s; (**g**) 50~100 s; (**h**) 5~100 s.

For example, the spatial distribution of the anomaly in 2016 shows that the Cangwu Guangxi M5.4 earthquake (yellow circle with green edge) was located in the polarization high-value area in the full-frequency band (Figure 2a–d). Similarly, the anomalous spatial distribution in June 2018 corresponds well with that of the Mojiang 5.9 earthquake (yellow circle with green edge) (Figure 2e–h). In addition to the 5~25 s frequency band, both the 25~50 s and 50~100 s frequency bands highlight the high-value anomalous areas. Hence, by refining the frequency bands, the areas of significant anomalies in the 25–50 and 50–100 s bands are narrowed, and most of the earthquakes occur in and around anomalous regions. However, in the process of analysis, there are some cases in which the anomalous area has high values in the full-frequency band but no corresponding earthquakes, and the related reasons need to be further explored.

**Figure 3:**
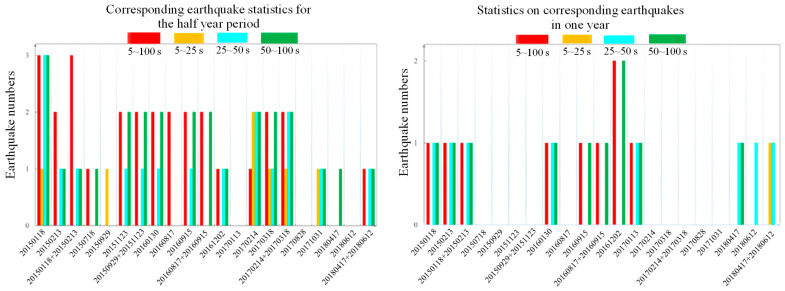
Histogram comparing the number of earthquakes with the number of polarization anomalies in different frequency bands. The left panel shows the corresponding earthquake numbers for the half-year periods of anomalies in different frequency bands; the right panel shows the corresponding earthquake numbers for the half-year periods of anomalies in different frequency bands.

By counting the high-value anomalous areas in different frequency bands of polarization and comparing them with the full-frequency band, we found that the anomalous areas can be better focused, especially with the help of data from the 25–50 and 50–100 s bands (Figure 3). By refining the frequency bands, we can effectively narrow the distribution range of polarized high-value anomalies to certain seismic events, filter out some of the effects of short-period disturbances, and improve the utilization rate of the data for earthquake forecasting. Through this comparison, we noticed that the data with a frequency band of 50~100 s are relatively better than those with a frequency band of 5~50 s when predicting M > 5.5 earthquakes within one year in western China.

## 4. Discussion

Observations and analyses have shown that when the signal originates from the high-altitude ionosphere (magnetosphere), the ULF magnetic field polarization value *Y_zh_* is usually less than 1, whereas the ULF magnetic field polarization value *Y_zh_* from seismogenic regions is ≥1; therefore, the polarization value is considered the key to differentiating between geomagnetic radiation originating from the ionosphere (magnetosphere) and seismic magnetic radiation originating from subsurface rocks (e.g., [18,33,34,35]). In addition, precursory anomalies are usually indicated by high vertical intensity polarization ratio values (*Y_zh_* ≥ 1), while the postearthquake effects of geomagnetic observations are usually distinguished by the reduction in (recovery of) the vertical intensity polarization ratio to a normal range (*Y_zh_* < 1). Furthermore, we used the noise reduction process of the China Geomagnetic Observatory Network to eliminate ULF Pc1 geomagnetic pulsations through several indices to evaluate the reference background noise. The indices are designed according to the distribution characteristics of international magnetic quiescence and disturbance days (e.g., [19,36,37]). By comparing the reference background noise during the midnight period (with the most active excited geomagnetic wave noise) with the reference background noise during the quiescence period by using the first-order difference method, the indices effectively reduced most of the noise from excited geomagnetic waves (e.g., [32]).

Rock mechanics pressure tests have shown that the conductivity of a rock increases when it is close to rupture, and according to the principle of electromagnetic induction, a change in underground conductivity will inevitably cause a change in the geomagnetic field [20,38]. The complex processes underlying earthquakes are fundamentally linked to tectonic activities involving mechanical processes such as mutual friction, extrusion, deformation, and fault rupture. This complex interplay does not confine itself to mere points but unfolds across lines and surfaces, creating a multifaceted process of seismic activity that extends well beyond localized phenomena (e.g., [39,40,41]). The mechanical interactions that occur between faults during an earthquake, including friction, extrusion, deformation, and rupture, are not silent. They induce temporary currents in the vicinity of the seismic area, leading to diverse electromagnetic anomalies. The complexity of the induced magnetic field is further highlighted by the intermittent and continuous high-value patterns observed in geomagnetic vertical intensity polarizations (e.g., [42]). These patterns suggest that the electromagnetic signals generated by these mechanical interactions are inherently random and discontinuous, posing challenges for observation stations near the epicenter in reliably capturing these signals (e.g., [42]).

Comprehensive analysis revealed that the anomalies at high geomagnetic vertical intensity polarizations are not related to the exogenous field or to the subsurface electromagnetic signals. When the geological block stress environment changes and displacement occurs or is about to occur, a temporary induction current will be generated nearby. Affected by the lateral nonhomogeneity of the underground medium, the electromagnetic radiation signal attenuates differently, resulting in various patterns of change in different frequency ranges. The pursuit of a more nuanced understanding of seismic phenomena continues to drive research across multiple disciplines (e.g., [43,44]). Future advancements in sensor technology, computational modeling, and data analysis are poised to offer new insights into the precursors of seismic events. Such advancements could revolutionize the field of earthquake prediction, providing the necessary tools to decipher the complex signals associated with tectonic movements.

In the current geomagnetic vertical intensity polarization analysis in China, the 5~100 s frequency band information is generally selected as the full band for prequake anomaly studies. However, these frequency bands within the range of 5~100 s correspond to different skinning depths. Since the resistivity at the seismic source cannot be obtained accurately, we replace it with the resistivity of sedimentary rocks (100 Ωm). Based on the skinning effect, the 5~100 s band corresponds to depths of 28~126 km, that is, 5~25 s corresponds to depths of 28~63 km, 25~50 s corresponds to depths of 63~89 km, and 50~100 s corresponds to depths of 89~126 km. To obtain a valuable understanding of the ability of observation data and to make practical and organized earthquake predictions, we used three bands currently. Our objective is to cover a wider variety of signals to ensure that the analysis is feasible and interpretable. Future studies are needed to determine the benefits of using more frequency bands. In addition, a large amount of experimental research has demonstrated the emission of electromagnetic signals from rocks in the kHz-MHz frequency band, but the resolution of geomagnetic observations in the China Geomagnetic Observatory Network is 0.01 s; thus, these signals in the kHz-MHz frequency band are not analyzed in this study due to resolution limitations.

The research results in this study are based on the geomagnetic vertical intensity polarization results calculated from the 2015~2018 magnetic flux gate-second sampling data in mainland China, with a total of eleven groups of anomalies, including sixteen single-day anomalies and five superimposed anomalies, on which the frequency bands are redetailed to address the results, which may be of some reference value to subsequent earthquake prediction work. However, because the number of seismic cases and the accumulation of data are not sufficiently rich, the conclusions of this study are only the preliminary conclusions reached at present, and the information and seismic cases will be updated in the subsequent period to make the conclusions and results more objective and scientific.

Furthermore, the integration of geological, mechanical, and electromagnetic data could pave the way for innovative approaches to seismic monitoring. By synthesizing information from these diverse fields, researchers can develop more accurate models to predict seismic activity, potentially leading to breakthroughs in our ability to mitigate the impacts of earthquakes.

## 5. Conclusions

The existing analyses of geomagnetic vertical intensity polarization are all based on the 5~100 s frequency band without refinement of the partitioning process. Through this study, based on observations in western China, where fluxgate observation points are positioned in areas with frequent, densely distributed medium-strength earthquakes, we refined the frequency bands of geomagnetic vertical intensity polarization and recalculated the spatial and temporal evolution of geomagnetic disturbances before M > 5.5 earthquakes and obtained the following conclusions.

(1) The anomaly of geomagnetic vertical intensity polarization in the 25~50 s and 50~100 s bands can effectively narrow the estimated distribution range of the polarization high-value anomalies to certain seismic events.

(2) Based on the geomagnetic vertical intensity polarization results calculated from the 2015~2018 magnetic flux gate-second sampling data in mainland China, with a total of 11 groups of anomalies, the data with a frequency band of 50~100 s are relatively better than those with a frequency band of 5~50 s when predicting M > 5.5 earthquakes within one year in western China.

(3) Affected by the lateral nonhomogeneity of the underground medium, the electromagnetic radiation signal shows various patterns of change in different frequency ranges, which can be effectively utilized through the refinement of the frequency partitioning process.

## Figures and Tables

**Table 1:** Geomagnetic vertical intensity polarization frequency division anomaly statistics.

No.	Anomaly Date	Anomaly Station Percentage	Eq No.	Magnitude	Epicenter	Date	5~25 s	25~50 s	50~100 s
1	20150118	71.70%	1	5.8 8.2 6.5	Azuoqi Nepal Pishan Xinjiang	20150415 20150425 20150703	↓	↓	↓
2	20150213	40.74%					↓	↓	↑
	20150118 + 20150213						↓	↓	↓
3	20150718	38.46%	2	6.5	Pishan Xinjiang	20150703	↓	↑	↓
4	20150929	24.14%	3	7.7 6.4	Tajikistan Menyuan Qinghai	20151207 20160121	↓	↑	↓
5	20151123	67.24%					↓	↓	↓
	20150929 + 20151123						↓	↓	↑
6	20160130	51.52%	4	6.4 5.4	Kyrgyzstan Cangwu Guangxi	20160626 20160731	↓	↓	↑
7	20160817	45.31%	5	6.3 6.8 6.2	Maidu Qinghai Aketedu Xinjiang Hutubi Xinjiang	20161017 20161125 20161208	↓	↓	↓
8	20160915	52.38%					↓	↓	↓
	20160817 + 20160915						↓	↑	↑
9	20161202	43.55%	6	6.2	Hutubi Xinjiang	20161208	↓	↓	↑
10	20170113	20.63%	7	6.2	Hutubi Xinjiang	20161208	↓	↑	↑
11	20170214	22.58%	8	7.1 6.6	Jiuzhaigou Sichuan Jinghe Xinjiang	20170808 20170809	↑	↑	↑
12	20170318	45.31%					↓	↑	↑
	20170214 + 20170318						↑	↑	↑
13	20170828	47.69%	9	6.6	Jinghe Xinjiang	20170809	↓	↑	↑
14	20171031	29.69%	10	6.9	Miling Tibet	20171118	↑	↑	↑
15	20180417	28.99%	11	5.9	Mojiang Yunnan	20180908	↓	↑	↑
16	20180612	43.94%					↓	↓	↑
	20180417 + 20180612						↓	↑	↑

## Data Availability

Data are available in the figures and tables of the manuscript or from the authors upon request.
